# Biopsychosocial Correlates of Presence and Intensity of Pain in Adolescents With Inflammatory Bowel Disease

**DOI:** 10.3389/fped.2020.00559

**Published:** 2020-09-08

**Authors:** Lexa K. Murphy, Jason D. Rights, Amanda Ricciuto, Peter C. Church, Sara Ahola Kohut

**Affiliations:** ^1^Center for Child Health, Behavior and Development, Seattle Children's Research Institute, Seattle, WA, United States; ^2^Department of Psychology, University of British Columbia, Vancouver, BC, United States; ^3^Division of Gastroenterology, Hepatology, and Nutrition, The Hospital for Sick Children, Toronto, ON, Canada; ^4^SickKids Research Institute, Toronto, ON, Canada; ^5^Department of Paediatrics, University of Toronto, Toronto, ON, Canada; ^6^Department of Psychiatry, University of Toronto, Toronto, ON, Canada

**Keywords:** inflammatory bowel disease, pediatric, pain, psychosocial, anxiety, depression, parents

## Abstract

**Background:** There is growing consensus that pain in pediatric inflammatory bowel disease (IBD) is not fully explained by disease-related processes. However, previous studies have largely measured individual biological, psychological, or social risk factors for pain in isolation. Further, not all youth with IBD presenting to clinic will report presence of pain, and those who do vary in their reports of pain intensity. This study therefore extends prior research by determining biopsychosocial correlates of both presence and intensity of pain in adolescents with IBD, in order to inform targeted pain management intervention approaches.

**Methods:** Adolescents with IBD followed at SickKids, Toronto, and their parents were consecutively enrolled from outpatient clinic. IBD characteristics (diagnosis, time since diagnosis, patient-reported disease activity) were collected. Adolescents reported on current pain (NRS-10), internalizing symptoms (Strengths and Difficulties Questionnaire), and pain catastrophizing (Pain Catastrophizing Scale-Child). Parents reported on protective responses to child pain (Adult Responses to Child Pain) and pain catastrophizing (Pain Catastrophizing Scale-Child). Hurdle models were conducted to examine predictors of presence and intensity of pain in the same model. Biological (patient-reported disease activity, IBD diagnosis subtype, illness duration), psychological (internalizing symptoms, pain catastrophizing), and social (parent pain catastrophizing, parent protective responses) factors were entered as predictors, adjusting for age and sex.

**Results:** Participants included 100 adolescents (12–18; *Mean* = 15 years) with IBD (60% Crohn's Disease, 40% Ulcerative Colitis or IBD-unclassified) and 76 parents. The majority of the sample was in clinical remission or reported minimal symptoms. Half of participants reported no current pain; for those reporting pain, intensity ranged 1–7 (*M* = 3.43, SD = 1.98). Disease activity (OR = 53.91, *p* < 0.001) and adolescent internalizing symptoms (OR = 7.62, *p* = 0.03) were significant predictors of presence of pain. Disease activity (RR = 1.37, *p* = 0.03) and parent protective responses (RR = 1.45, *p* = 0.02) were significant predictors of intensity of pain.

**Conclusions:** Results suggest that the experience of pain in pediatric IBD is biopsychosocially determined. Patient-reported disease activity and internalizing symptoms predicted presence of pain, while disease activity and parent protective responses predicted intensity of pain. While medical intervention in pediatric IBD is focused on disease management, results suggest that depression/anxiety symptoms as well as parent protective responses may be important targets of pain management interventions in pediatric IBD.

## Introduction

Pain is a common symptom of inflammatory bowel disease, with the majority of patients reporting pain at initial diagnosis and during recurrence (1). Pain is associated with impaired psychological and physical health outcomes and greater healthcare expenses in youth with IBD, over and above the influence of disease severity (2–5). While medical intervention in pediatric IBD is focused on disease management (i.e., the escalation of pharmacological intervention), there is increasing concern that pain management has been neglected (6, 7). For example, pain continues to be a persistent problem in the time that it takes for medication to be optimized, and emerging research indicates that a significant subset of youth still report pain even during disease remission (2, 5, 8, 9). While non-pharmacological interventions for pediatric pain exist and demonstrate efficacy in reducing pain and disability (10), more information about correlates of pain in adolescents with IBD is needed in order to develop tailored pain management interventions.

There is growing consensus that pain in pediatric IBD is not fully explained by disease-related processes and instead is biopsychosocially determined (6, 11, 12). Disease-related processes that contribute to pain include acute inflammation, strictures, bowel obstruction, and dysmotility, as well as visceral hypersensitization (1, 6). Previous studies of pain in pediatric IBD have often measured disease activity with indices that include subjective pain ratings, however this may artificially inflate associations between disease activity and pain (11). In recent years, patient-reported outcomes (PROs) have garnered significant attention and are increasingly used in the study of IBD to reflect disease activity, while incorporating the patient's experience of disease (13). Illness duration and IBD type (Crohn's Disease and Ulcerative Colitis) have been examined less frequently as correlates of pain, and previous studies have found inconsistent results (4, 11, 14). Psychological correlates of pain in pediatric IBD include internalizing symptoms (i.e., anxiety and depression) (2, 4, 14–16) as well as pain-specific cognitive factors such as pain catastrophizing (16, 17) (i.e., magnifying or exaggerating the threat or seriousness of painful sensations (18). Social correlates of pain in pediatric IBD have been examined less frequently and greater understanding is needed. Previously, researchers have focused primarily on the social influence of parents (19). Parent protective responses [e.g., letting children stay home from school when they are in pain, giving special treats when their child has pain (20)] and parent catastrophizing about child pain have been examined in two small trials with mixed findings (5, 21). However, previous pediatric IBD studies have measured individual risk factors for pain in isolation or controlled for specific factors, but to date no study has measured biological, psychological, and social factors together in the same model.

Measuring pain in IBD has also proved challenging, given that not all youth with IBD presenting to clinic will report presence of pain, and those who do vary in their reports of pain intensity. Prior studies have focused exclusively on measuring either presence of pain [e.g., (22, 23)] or intensity of pain [e.g., (21, 24)]. Focusing on pain intensity ratings provides important information about factors that are related to the experience of pain, but presents the challenge of positively skewed, zero-inflated distributions of pain scores. Additionally, it is possible that certain factors may exacerbate pain (i.e., may predict increased intensity), but would not necessarily be related to whether or not one has any pain (i.e., may not predict the presence of pain). On the other hand, though dichotomizing pain scales to presence vs. absence allows researchers to determine important potential factors that do predict pain presence, it removes important contextual information about the intensity of that pain (25) and distorts inferential testing in numerous ways (26). In addition, chronic pain researchers have found that pain intensity scales not only capture information about intensity, but also reflect perceptions about pain interference and beliefs about pain [for example, pain unpleasantness (27–29)], suggesting that pain intensity may be more closely linked to psychosocial factors than biological factors. This study therefore extends prior research by determining biopsychosocial correlates of both *presence* as well as *intensity* of pain together in the same model in a way that avoids the issues inherent to zero-inflated or forcibly dichotomized data.

## Aims and Hypotheses

The primary aim of this study is to determine biopsychosocial correlates of presence and intensity of pain in adolescents with IBD. When examining all factors simultaneously in the same model, we expect that biological factors such as disease activity will predict presence of pain, while psychosocial factors will predict intensity of pain. Given the dearth of studies that have examined biopsychosocial factors simultaneously in pediatric IBD, our exploratory aim is to determine correlations among these factors in youth with IBD.

## Methods

### Participants and Procedure

This study was part of a larger cross-sectional study designed to examine adolescent and family adjustment to IBD. This study was approved by the research ethics board at the Hospital for Sick Children (#1000051104). Adolescents diagnosed with IBD and their primary caregivers were consecutively invited to participate; enrollment occurred between 2016 and 2017. Inclusion criteria were age 12–18 years, proficient in speaking and reading English, and no significant cognitive impairment or major co-morbid illnesses. Eligible patients were invited to the study at the time of their scheduled IBD clinic visit.

### Measures

#### Sociodemographic Factors

Adolescents and parents self-reported age, sex, and race/ethnicity from options provided (instructed to “check all that apply”).

#### Pain

Adolescents self-reported average IBD pain intensity over the last 7 days on a 0–10 numerical ratings scale [NRS-11 (30)]. The NRS-11 has been well-validated for use with adolescents and is frequently employed by pediatric gastroenterologists during routine clinic visits (6).

#### Biological/Disease Factors

Adolescents self-reported IBD diagnosis (Crohn's Disease or Ulcerative Colitis/IBD-unclassified), duration (from time of diagnosis) of illness, current medications (checked and confirmed by a nurse), presence of ostomy, and current stool frequency and characteristics. IBD disease activity was measured using the disease activity item from the Patient-Reported Outcome-Based Evaluation [PROBE (31)], which is a 5-point Likert Scale that assessed IBD activity over the past week (0 = *remission*, 1 = *minimal symptoms*, 2 = *mildly active*, 3 = *moderately active*, 4 = *severely active*). In addition, to support the face validity of this measure, we examined how stool characteristics (frequency, consistency, blood) varied with changes in this self-reported activity score.

#### Psychological Factors

Adolescents self-reported internalizing symptoms (anxiety and depression symptoms) with the Strengths and Difficulties Questionnaires-Emotional Problems subscale (32). This subscale consists of 5 items assessing frequency/intensity of internalizing symptoms over the past 6 months (e.g., “I worry a lot;” “I am often unhappy, depressed, and tearful”) on a 3-point Likert Scale (1 = *not true*, 3 = *certainly true*) and the total score (0–15) is reported. Pain catastrophizing was assessed with the Pain Catastrophizing Scale-Child [PCS-C (33)]. Thirteen items assess catastrophic thoughts and feelings about pain (e.g., “When I am in pain, I wonder whether something serious may happen”) on a 5-point Likert Scale (0 = *not at all*, 4 = *extremely*) and the total score (0–65) is reported.

#### Parent Factors

Parent pain catastrophizing was assessed with the Pain Catastrophizing Scale-Parent [PCS-P (34)]. Thirteen items assess catastrophic thoughts and feelings about their child's pain (e.g., “When my child is in pain, I become afraid that the pain will get worse”) on a 5-point Likert Scale (0 = *not at all*, 4 = *extremely*) and the total score (0–65) is calculated. Parents reported their responses to children's pain with the Adult Responses to Child Pain-Protect subscale. This 13-item subscale assesses parental protective responses to child's pain (e.g., “When your child has a stomachache or abdominal pain, how often do you let him/her stay home from school?”) on a 5-point Likert Scale (0 = *never*, 4 = *always*) and a mean score (0–4) is calculated.

### Statistical Analyses

Skewness was <1.5 and kurtosis (κ) was <2 for all variables. Spearman's and Pearson's correlations were conducted to examine correlations among biopsychosocial variables. Exploratory analyses were conducted to examine differences between the group reporting no pain and the group reporting any pain with Student's *t*, Mann-Whitney *U*, and Chi-squared tests.

To examine multivariable predictors of both the presence of pain and the intensity of pain in a single analysis, we used a hurdle model, which naturally accounts for the zero-inflation and positive skew of average pain ratings. Hurdle models involve two sub-models that explore two outcomes. The first is a logistic regression predicting zero vs. non-zero values (presence of pain). The second is a non-zero truncated Poisson regression for the distribution of non-zero values predicting pain ratings (intensity of pain). Predictors were the same across models and included biological (patient-reported disease activity, IBD diagnosis subtype, illness duration), psychological (internalizing symptoms, pain catastrophizing) and social (parent pain catastrophizing, parent protective responses) factors while adjusting for sociodemographic factors (age and sex). Psychological factors were transformed to *z*-scores (calculations based on means and standard deviations from the current sample) before being entered in hurdle models to aid in interpretation. Odds ratios (for the logistic regression sub-model), risk ratios (for the Poisson regression sub-model), and associated 95% confidence intervals were calculated for each predictor. In addition, sensitivity analyses were conducted with the subgroup reporting remission or minimal symptoms. Descriptive statistics were performed in SPSS v19.0 (35). Hurdle models were calculated using the hurdle function in the PCSL package for R (36).

## Results

### Preliminary Analyses

In total, 100 adolescents and 76 primary caregivers gave informed consent to participate in the study. Descriptives for sociodemographic and clinical variables are reported in Table 1. The majority of the sample was diagnosed with CD (60% CD, 40% UC or IBD-Unclassified) and average illness duration was 2 years. Participants reported a range of disease activity over the past week, though the majority had no to mild symptoms; 38% were in remission, 33% reported minimal symptoms, 18% reported mildly active, 6% reported moderately active, and 5% reported severely active.

**Table 1:** Descriptives for primary study variables.

	**M (SD) or *N* (%)**	**Range**
**Sociodemographics**		
Adolescent age (years)	15.1 (1.5)	12–18
Adolescent sex	54 (54%) Male; 46 (46%) Female	–
Parent age (years)	46.1 (6.0)	32–58
Parent sex	15 (20%) Male; 38 (80%) Female	–
Adolescent race/ethnicity	58 (58%) White 22 (22%) Asian 4 (4%) Middle eastern 1 (1%) Black 13 (13%) Mixed 2 (2%) Not reported	–
**Disease factors**		
IBD type	60 (60%) Crohn's disease 40% Ulcerative colitis/IBD-unclassified	–
Years since IBD diagnosis	2.3 (2.4)	0–10
Disease activity in past week	43 (43%) Remission 38 (38%) Minimal symptoms 9 (9%) Mildly active 3 (3%) Moderately active 5 (5%) Severely active 2 (2%) Not reported	–
Medication	53 (53%) Biologic 25 (25%) Aminosalicylate/sulfasalazine 46 (46%) Immunomodulator 30 (30%) Antibiotic	–
Ostomy	4 (4%) Yes	–
Average adolescent pain^a^	1.6 (2.2)	0–7
**Adolescent factors**		
Internalizing symptoms^b^	10.1 (2.1)	6–15
Pain catastrophizing^c^	26.8 (11.2)	6–52
**Parent factors**		
Pain catastrophizing^c^	26.5 (10.6)	0–52
Protective responses to child pain^d^	1.9 (0.7)	0.7–4.0

M, Mean; SD, Standard deviation; N = 100 for Child variables, N = 78 for Parent variables

a *NRS-10 Range 0–10*;

b *Strengths and Difficulties Questionnaire-Emotional Problems Range 0–15*;

c *Pain Catastrophizing Scale-Children and Parent Scales Range 0–65*;

d *Adult Responses to Child Symptoms-Protect Range 0–4; Disease activity measured with the PROBE*.

**Figure 1:**
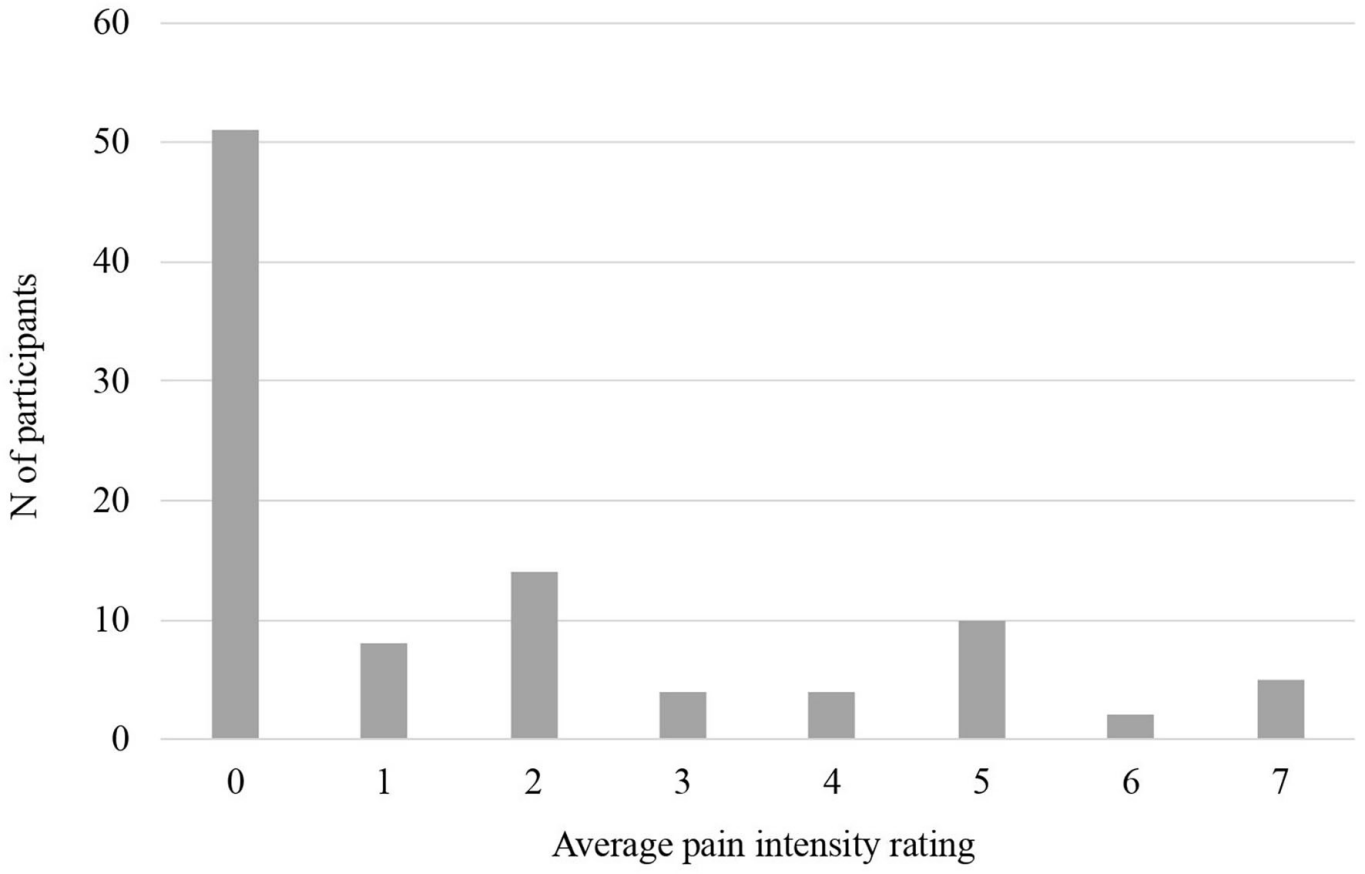
Pain intensity ratings.

Approximately half (52%) of participants reported 0 for average pain intensity over the past week. For those reporting pain, intensity ranged 1–7 (M = 3.43, SD = 1.98). Eight percent reported average pain intensity of 1, 14% reported 2, 4% reported 3, 4% reported 4, 10% reported 5, 2% reported 6, and 5% reported 7. See Figure 1. Differences between groups reporting pain vs. no pain were examined across key predictor variables and results are depicted in Table 2. Youth reporting presence of pain reported significantly worse disease activity, internalizing symptoms, and pain catastrophizing. When pain was examined continuously, female adolescents reported more intense pain than male adolescents (*M*_female_ = 2.07, *SD*_*f*__emale_ = 2.40; *M*_male_ = 1.13, *SD*_male_ = 1.83, *p* = 0.03). None of the primary study variables differed significantly by parent sex (*p*'s > 0.47). Disease activity ratings increased significantly in accordance with stool symptoms (increasing in number, unformed consistency, and with blood). See Supplemental Materials.

**Table 2:** Differences between groups reporting no pain vs. any pain.

	**Pain absent (*N* = 52) M (SD) or *N* (%)**	**Pain present (*N* = 48) M (SD) or *N* (%)**	***p*-value**
**Sociodemographics**			
Age	15.2 (1.6)	15.0 (1.5)	0.62
Sex	55% Female	35% Female	0.06
**Disease factors**			
Disease activity in past week	67% Remission	7% Remission	<0.001
Illness duration in years	2.4 (2.3)	2.2 (2.6)	0.62
IBD diagnosis subtype	60% Crohn's disease	60% Crohn's disease	0.85
**Adolescent factors**			
Internalizing symptoms	9.5 (1.8)	10.8 (2.1)	<0.01
Pain catastrophizing	23.7 (12.2)	28.9 (11.0)	0.05
**Parent factors**			
Pain catastrophizing	26.2 (10.9)	27.55 (9.9)	0.57
Protective responses to child pain	1.9 (0.7)	1.97 (0.7)	0.70

*Differences between groups determined by t-tests for age, Illness duration, and adolescent and parent factors; by Chi-squared tests for sex and IBD diagnosis; and by Mann-Whitney U-test for disease activity. N = 76 for analyses involving parent variables, N = 100 for analyses involving adolescent variables*.

### Correlational Analyses

Correlations among biopsychosocial variables are reported in Table 3. Disease activity was significantly, positively correlated with adolescent internalizing symptoms and pain catastrophizing (*p* = 0.001, *p* = 0.007, respectively). Illness duration was significantly, positively correlated with internalizing symptoms (*p* = 0.03). Adolescent internalizing symptoms and adolescent pain catastrophizing were significantly, positively correlated (*p* < 0.001). Parent and adolescent pain catastrophizing were also significantly, positively correlated (*p* = 0.001).

**Table 3:** Correlations among biopsychosocial factors.

	**1**	**2**	**3**	**4**	**5**	**6**
1. Disease activity^a^	–					
2. Illness duration	0.02	–				
3. Age	0.08	−0.04	–			
4. Adolescent internalizing Sx	0.34^**^	0.24^*^	0.06	–		
5. Adolescent pain catastrophizing	0.29^**^	−0.12	0.12	0.37^***^		
6. Parent protective responses	0.03	0.06	−0.12	−0.07	−0.06	
7. Parent pain catastrophizing	−0.07	−0.06	−0.02	0.11	0.37^**^	0.21

* *p < 0.05*,

** *p < 0.01*,

*** *p < 0.001; N = 98 for Child variables, N = 78 for Parent variables*.

a *Spearman's R was computed for all correlations with Disease Activity; Pearson's r was computed for all other correlations; Sx, Symptoms; disease activity measured with the PROBE; Internalizing symptoms (anxiety/depression symptoms) measured with the Strengths and Difficulties Questionnaire; pain catastrophizing measured with the Pain Catastrophizing Scale-Child and Pain Catastrophizing Scale-Parent; protective responses to child pain measured with the Adult Response to Child Pain—Protect Subscale. N = 76 for correlations involving parent variables, N = 100 for correlations involving adolescent variables*.

### Predicting Presence and Intensity of Pain

In the hurdle model predicting pain, biological (patient-reported disease activity, IBD diagnosis subtype, illness duration), psychological (internalizing symptoms, pain catastrophizing), and social (parent pain catastrophizing, parent protective responses) factors were entered as predictors while adjusting for sociodemographic factors (age and sex). See Table 4. In the logistic regression sub-model predicting presence of pain, only disease activity and adolescent internalizing symptoms were significant predictors. The odds of having pain vs. no pain increases by a factor of 54 for each unit increase in disease activity (OR = 53.91; 95% CI 6.62–438.79; *p* < 0.001). The odds of having pain vs. no pain increases by a factor of 8 for each standard deviation increase in adolescent internalizing symptoms (OR = 7.62; 95% CI 1.27–45.76; *p* = 0.03). In the Poisson regression sub-model predicting intensity of pain, only disease activity and parent protective responses were significant predictors. Predicted pain intensity increases by a factor of 1.37 for each unit increase in disease activity (RR = 1.37; 95% CI 1.02–1.83; *p* = 0.03). The predicted pain intensity increases by a factor of 1.45 for each standard deviation increase in parent protective responses (RR = 1.45; 95% CI 1.07–1.96; *p* = 0.02).

**Table 4:** Hurdle regression model predicting adolescent pain.

	**B (SE)**	**Odds ratio^d^ [95% CI]**	***P***
**LOGISTIC REGRESSION PREDICTING PRESENCE OF PAIN**			
Intercept	−19.15 (9.51)	0.00 [0.00, 0.60]	0.04
Age	−0.13 (0.32)	0.88 [0.46, 1.65]	0.68
Sex	1.91 (1.35)	6.76 [0.47, 96.27]	0.16
**Disease activity**	**3.99 (1.07)**	**53.91 [6.62, 438.79]**	**<0.001**
IBD diagnosis Subtype^a^	0.79 (1.02)	2.21 [0.30, 16.40]	0.44
Illness duration^b^	−0.32 (0.02)	0.97 [0.94, 1.00]	0.06
**Adolescent internalizing Sx^c^**	**2.03 (0.92)**	**7.62 [1.27, 45.76]**	**0.03**
Adolescent pain catastrophizing^c^	−0.49 (0.55)	0.62 [0.21, 1.79]	0.37
Parent protective responses^c^	0.29 (0.45)	1.34 [0.55, 3.25]	0.52
Parent pain catastrophizing^c^	0.52 (0.63)	1.67 [0.50, 5.81]	0.42
	**B (SE)**	**Risk ratio^e^ [95% CI]**	***P***
**TRUNCATED POISSON REGRESSION PREDICTING INTENSITY OF PAIN**			
Intercept	−0.95 (2.48)	0.39 [0.00, 49.94]	0.70
Age	−0.12 (0.08)	0.86 [0.75, 1.04]	0.14
Sex	0.33 (0.39)	1.39 [2.98, 0.65]	0.44
**Disease activity**	**0.31 (0.15)**	**1.37 [1.02, 1.83]**	**0.03**
IBD diagnosis subtype	0.12 (0.27)	1.13 [0.66, 1.93]	0.66
Illness duration^b^	0.00 (0.00)	1.00 [1.00, 1.01]	0.21
Adolescent internalizing Sx^c^	0.15 (0.16)	1.16 [0.85, 1.60]	0.35
Adolescent pain catastrophizing	0.07 (0.16)	1.07 [0.79, 1.46]	0.66
**Parent protective responses^c^**	**0.37 (0.15)**	**1.45 [1.07, 1.96]**	**0.02**
Parent pain catastrophizing^c^	0.05 (0.13)	1.06 [0.82, 1.36]	0.67

*Sx, Symptoms; disease activity measured with the PROBE; Internalizing symptoms (anxiety/depression symptoms) measured with the Strengths and Difficulties Questionnaire; pain catastrophizing measured with the Pain Catastrophizing Scale-Child and Pain Catastrophizing Scale-Parent; protective responses to child pain measured with the Adult Response to Child Pain—Protect Subscale*.

a *Reference Group = Crohn's Disease*;

b *Illness duration in months*;

c *Psychosocial variables entered as z-scores*;

d *exponentiated coefficients [exp(B)] represent the estimated factor by which the odds of presence of pain changes for each unit increase in the predictor*;

e *exponentiated coefficients [exp(B)] represent the estimated factor by which the expected pain intensity increases for each unit increase in the predictor. N = 76 for all analyses*.

Additional sensitivity analyses were conducted by re-running the hurdle model with the subgroup that reported remission or minimal symptoms, and disease activity was removed as a predictor. The overall pattern of significance was very similar to that of the full model. In the logistic regression sub-model predicting presence of pain, only adolescent internalizing symptoms was a significant predictor (OR = 10.29, *p* = 0.002). In the Poisson regression sub-model predicting intensity of pain, only parent protective responses (RR = 2.08, *p* = 0.026) and female sex (RR = 7.19, *p* = 0.031) were significant predictors.

## Discussion

Results from this study suggest that the experience of pain in IBD is related to patient and parent-reported biological, psychological, and social factors. This study presents a novel analytic approach that examines both intensity and presence of pain simultaneously in the same analysis, which may provide an important model for other chronic health conditions that are characterized by diversity in pain presentations. Findings from this sample indicate that disease activity and adolescent internalizing symptoms are significant correlates of presence of pain, while disease activity and parent behavior are significant correlates of pain intensity.

Our hypothesis that biological factors would predict presence of pain while psychosocial factors would predict intensity of pain was partially supported. In our regression analyses, disease activity was a strong predictor of presence of pain, with the odds of having pain increasing by a factor of 54 for each unit increase in disease activity. In contrast, while disease activity was a significant predictor of pain intensity, the association was not as strong. Although it is unsurprising that disease activity would be related to pain in a chronic inflammatory condition such as IBD, this is the first study to parse out its impact on presence and intensity of pain separately. The disease activity score is an omnibus self-report and was significantly associated with increase in stool symptoms, supporting its validity as an activity measure. Although it is not as precise as composite disease indices, it represents an improvement over previous studies as it does not include subjective pain ratings in its measurement. Disease-related factors that may increase risk for pain include acute inflammation, strictures, and visceral hypersensitization (1, 6).

Interestingly, psychosocial factors were significant predictors of both presence and intensity of pain. Internalizing symptoms, which include symptoms of depression and anxiety, was a significant predictor of presence of pain, although the odds ratio was considerably smaller than disease activity. This is consistent with multiple previous studies linking depression and anxiety symptoms to abdominal pain in adult and pediatric IBD populations (11, 37). Previous research has suggested that internalizing symptoms may increase risk for pain by amplifying descending pain pathways and increasing pain signaling (38) or by increasing attention to pain (39). However, when examining intensity of pain in regression analyses, only parent protective responses was a significant psychosocial predictor. Although parent behavior has been studied frequently in chronic idiopathic pain populations (40), to our knowledge, this is the first time parent protective responses has been examined in relation to child pain in IBD. This finding suggests that parent responses to pain that include giving the adolescent special attention, limiting normal activities, and reducing responsibilities when they have pain may serve to increase the experience of pain in the setting of IBD.

It was surprising that neither parent nor adolescent pain catastrophizing was significantly related to adolescent pain in regression analyses. Pain catastrophizing describes magnifying or exaggerating the threat or seriousness of painful sensations (41). Mean levels in this sample are akin to youth with chronic pain conditions, and significantly higher than community samples of youth [*M* = 17, *SD* = 8 compared to *M* = 27, *SD* = 11 in this sample (33)]. When examining differences between youth reporting any pain vs. no pain, youth reporting pain also reported higher pain catastrophizing. This suggests that youth experiencing pain are also more likely to engage in catastrophizing, which is consistent with previous studies that found that catastrophizing was related to increased functional disability in youth with IBD (16, 17). In the context of pediatric IBD, pain catastrophizing may be driven by worries that pain is indicative of a worsening disease process. Although parent pain catastrophizing is linked to child pain in idiopathic pain populations (42), results from this study suggest that parent behavior, instead of parent thought patterns, is more closely linked to adolescent pain in IBD and may represent a more proximal intervention target.

Findings from exploratory analyses are also illuminating. Disease activity was significantly, positively related to adolescent internalizing symptoms and pain catastrophizing. This is consistent with prior research that has found that depression symptoms and disease activity are correlated cross-sectionally in IBD (43). This may also suggest that youth are more likely to catastrophize about pain when experiencing greater disease activity. During an IBD flare, pediatric patients may be told by physicians that pain is a sign of disease activity or recurrence and be cautioned to remain hypervigilant to pain (44). Given the self-report nature of disease activity in this study, we cannot entirely rule out that the reverse is true, such that increased internalizing symptoms and catastrophizing may increase perception of disease activity. The association between self-reported activity and other, less subjective symptoms of active disease (diarrhea, bloody stool) makes this less likely but longitudinal studies are needed to more definitively characterize the direction of the relationship among these variables.

This study has important clinical and research implications. While treating disease activity is a primary medical target for IBD, results suggest that psychosocial factors may also represent important targets for pain management even in patients with minimal disease activity seen in outpatient clinic visits. Previous studies have successfully tailored psychological interventions for adolescents with IBD (45–48), yet these trials did not explicitly target pain management or measure pain as an outcome. Psychological interventions for pediatric pain management exist and trials have demonstrated efficacy for reduction of pain and disability in both disease-related pain and idiopathic pain conditions (10). Given the increased psychological and physical health burden associated with pain in pediatric IBD (2, 4, 5), an important next step is tailoring these pain management interventions for youth with IBD. The results from this study suggest that adolescent internalizing symptoms and parent behavior may both be important targets. Intervening with parents around parent protective responses to child pain will require particularly sensitive clinical approaches. Unlike interventions in idiopathic pain conditions, which emphasize that parents need to reward youth for increased functioning, it is true that youth with IBD may be too ill to participate in school or daily activities when their IBD is flaring. Parents may require coaching around what types of signs and symptoms to attend to in IBD, while being careful not to inadvertently reinforce pain complaints or increase attention to pain itself.

Strengths of this study include thorough measurement of biopsychosocial factors, inclusion of both parent and adolescent reports, as well as a novel statistical approach that simultaneously considers both presence and intensity of pain. Limitations include measurement of disease activity by self-report, a relative reduction in power for analyses that involved parent variables (as fewer parents participated than adolescent patients), as well as the cross-sectional nature of this study. In addition, this sample was recruited from an outpatient clinic and a significant subset were in remission. Future studies would benefit from collecting data closer to time of diagnosis in samples with more active disease, and following patients over time. Such longitudinal studies would be able to better delineate biopsychosocial risk factors near the time of IBD diagnosis that may confer risk for pain over time, highlighting important targets for screening and prevention near diagnosis. Future studies may also consider examining social factors such as peer support and school functioning that may contribute to, or be impacted by, the experience of pain in pediatric IBD.

This study provides important information about biopsychosocial correlates of presence and intensity of pain in IBD. Disease activity and adolescent internalizing symptoms predicted presence of pain, while disease activity and parental behavior predicted pain intensity. In addition to medical management, this suggests that cognitive-behavioral treatment targeting psychosocial factors is needed in order to improve the care of youth with IBD.

## Data Availability Statement

The raw data supporting the conclusions of this article will be made available by the authors, without undue reservation to qualified researchers.

## Ethics Statement

The studies involving human participants were reviewed and approved by Hospital for Sick Children 1000051104. Written informed consent to participate in this study was provided by the participants' legal guardian/next of kin.

## Author Contributions

LM and SA contributed to the conception of the manuscript, data analysis plan, data analysis and interpretation, and writing of this paper. JR contributed to the data analysis plan, data analysis and interpretation, and writing. AR contributed to the data analysis plan, data interpretation, and writing. PC contributed to the data interpretation and writing. All authors contributed to the article and approved the submitted version.

## Conflict of Interest

The authors declare that the research was conducted in the absence of any commercial or financial relationships that could be construed as a potential conflict of interest.
