# Supplementary material for: Biopsychosocial Correlates of Presence and Intensity of Pain in Adolescents With Inflammatory Bowel Disease
Source: Front Pediatr. 2020 Sep 8;8:559. doi: 10.3389/fped.2020.00559 (PMC7506075; doi:10.3389/fped.2020.00559)

**Disease Activity by Number of Stools**

Mantel-Haenszel test of trend: χ²=18.24, *p*<.001


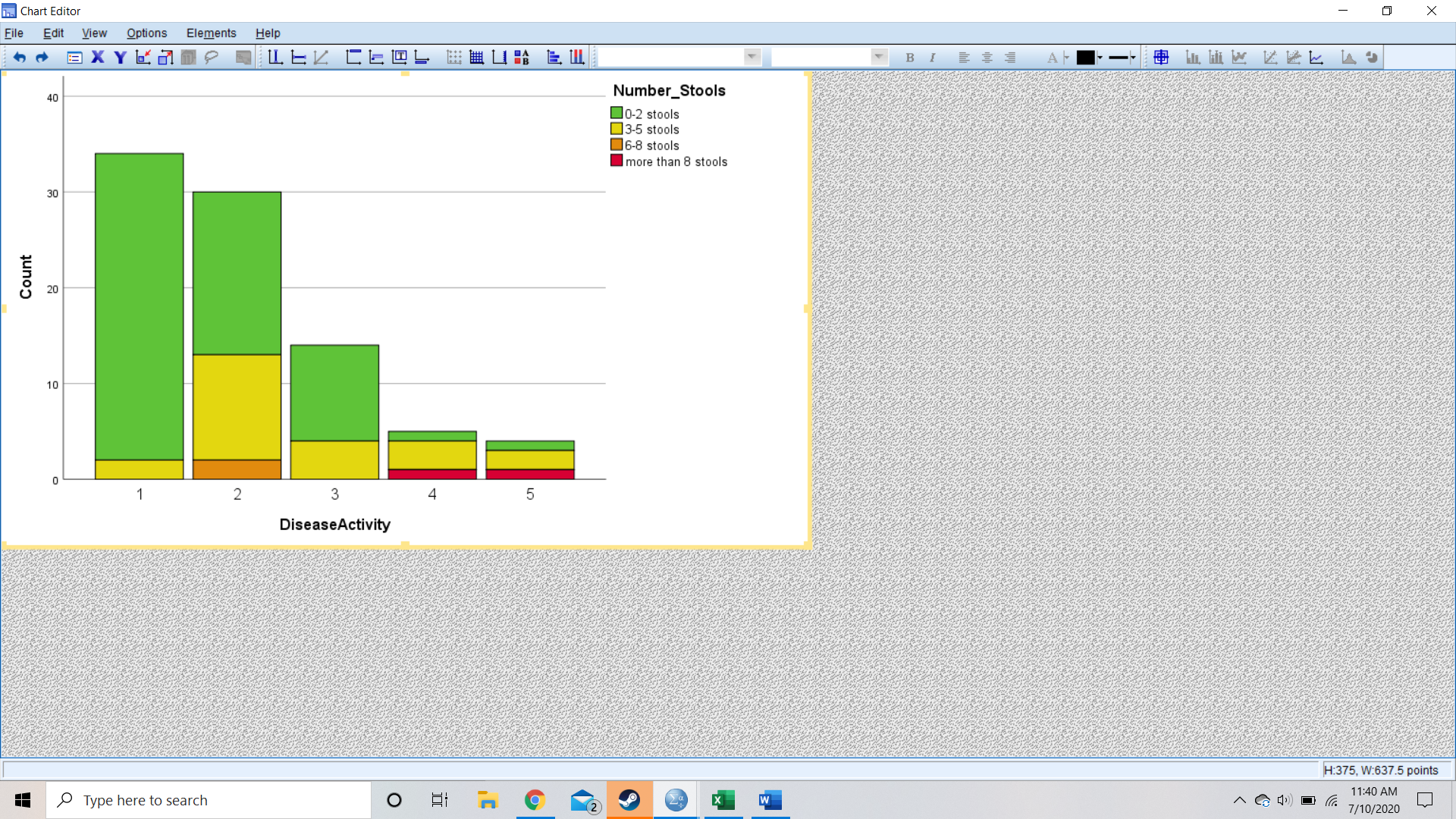


**Disease Activity by Consistency of Stools**

Mantel-Haenszel test of trend: χ²=18.575, *p*<.001


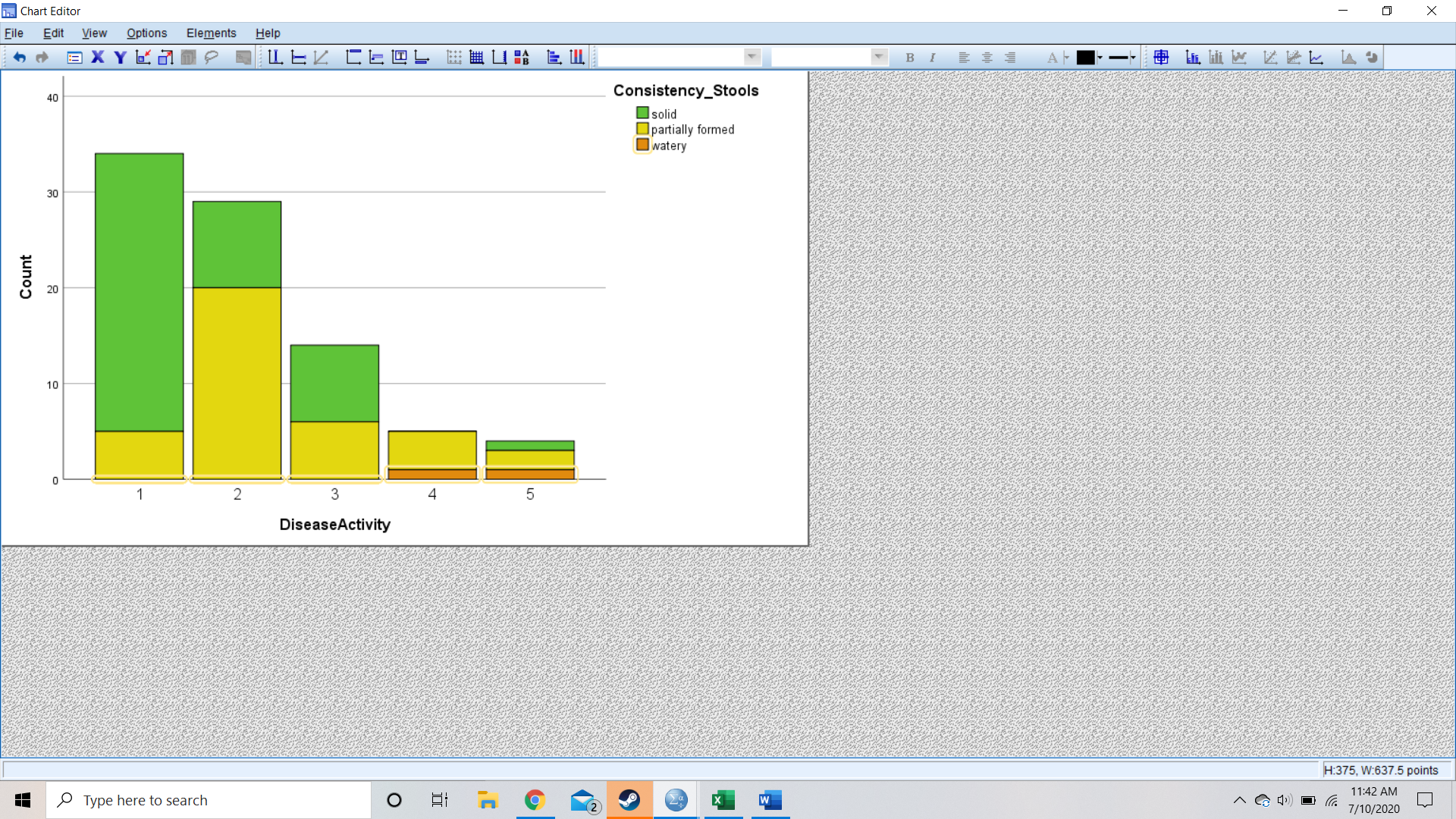


**Disease Activity by Blood in Stools**

Mantel-Haenszel test of trend: χ²=30.327, *p*<.001


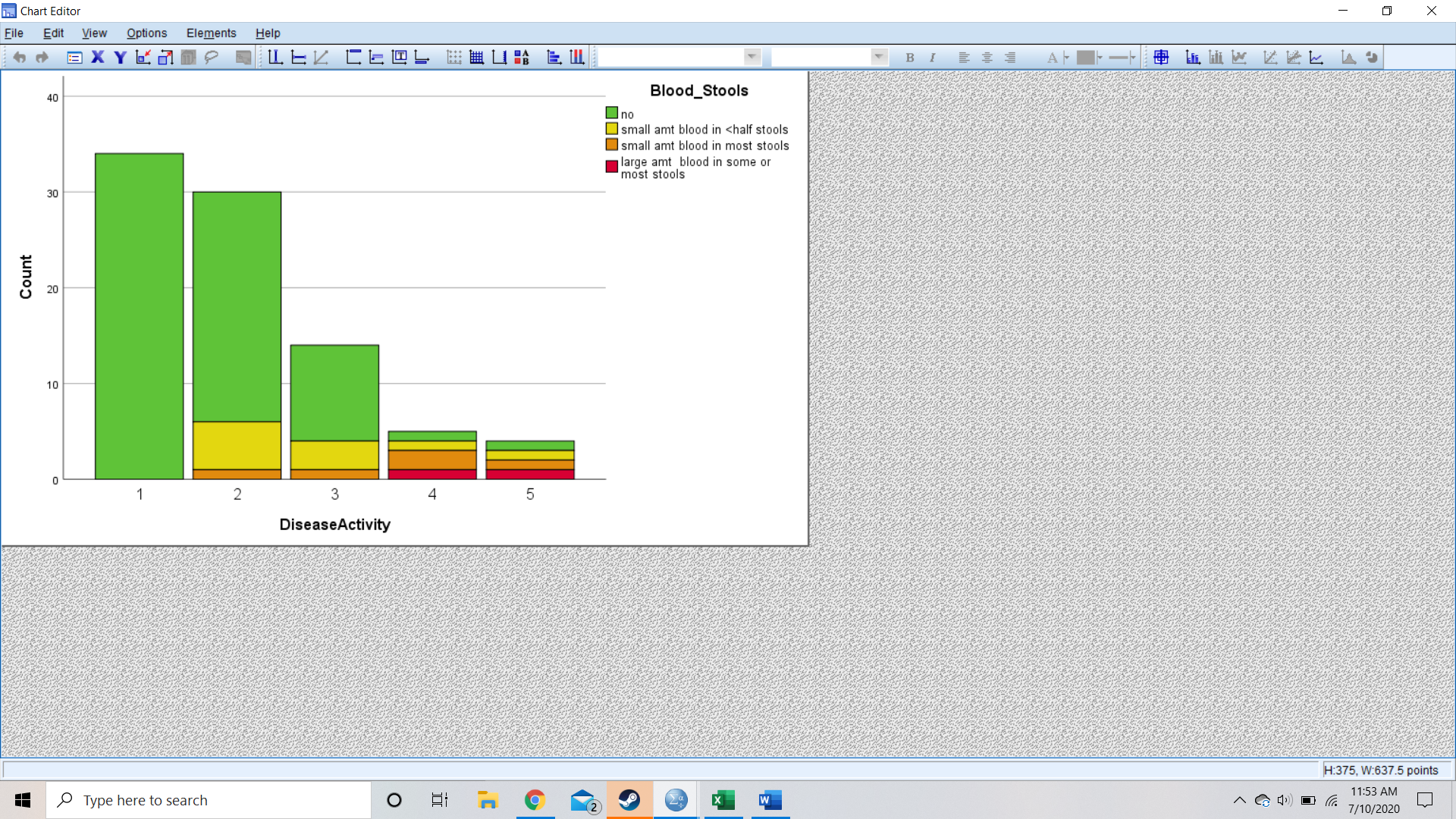

Supplement: Supplementary file 1 [file Data_Sheet_1.docx]
